# Circadian preference towards morningness is associated with lower slow sleep spindle amplitude and intensity in adolescents

**DOI:** 10.1038/s41598-017-13846-7

**Published:** 2017-11-03

**Authors:** Ilona Merikanto, Liisa Kuula, Tommi Makkonen, Róbert Bódizs, Risto Halonen, Kati Heinonen, Jari Lahti, Katri Räikkönen, Anu-Katriina Pesonen

**Affiliations:** 1 Department of Psychology and Logopedics, Faculty of Medicine, University of Helsinki, Helsinki, Finland; 2 National Institute for Health and Welfare, Helsinki, Finland; 3 Institute of Behavioural Sciences, Semmelweis University, Budapest, Hungary; 4 Epilepsy Centre, National Institute of Clinical Neurosciences, Budapest, Hungary; 5 Helsinki Collegium for Advanced Studies, Helsinki, Finland

## Abstract

Individual circadian preference types and sleep EEG patterns related to spindle characteristics, have both been associated with similar cognitive and mental health phenotypes. However, no previous study has examined whether sleep spindles would differ by circadian preference. Here, we explore if spindle amplitude, density, duration or intensity differ by circadian preference and whether these associations are moderated by spindle location, frequency, and time distribution across the night. The participants (N = 170, 59% girls; mean age = 16.9, SD = 0.1 years) filled in the shortened 6-item Horne-Östberg Morningness-Eveningness Questionnaire. We performed an overnight sleep EEG at the homes of the participants. In linear mixed model analyses, we found statistically significant lower spindle amplitude and intensity in the morning as compared to intermediate (P < 0.001) and evening preference groups (P < 0.01; P > 0.06 for spindle duration and density). Spindle frequency moderated the associations (P < 0.003 for slow (<13 Hz); P > 0.2 for fast (>13 Hz)). Growth curve analyses revealed a distinct time distribution of spindles across the night by the circadian preference: both spindle amplitude and intensity decreased more towards morning in the morning preference group than in other groups. Our results indicate that circadian preference is not only affecting the sleep timing, but also associates with sleep microstructure regarding sleep spindle phenotypes.

## Introduction

Individuals vary in their individual circadian timing of physiological functions and daily behavior activity patterns, such as sleep onset and awakening times, blood pressure, hormone secretion and core body temperature. Based on this variation, individuals differ by their circadian preference, those with earlier timed peaks in their physiological and behavioral functions named as morning-types, the later-timed named as evening-types, the intermediate-types falling in between these types in the timing of their physiological and behavioral functions^1–3^. Individual circadian preference is a biological character that is fairly unchangeable during adulthood^4^, with an heritability estimate of 47–49.7% based on cohort studies^5–7^.

Previous studies have reported consistent associations between circadian preference and sleep EEG patterns. Results from these studies indicate faster homeostatic sleep pressure dissipation based on slow-wave activity among morning preference as compared to evening preference group^8–11^, supporting a different homeostatic sleep regulation between morning and evening circadian preference groups. With regard to sleep stage architecture (e.g. percentages and duration of sleep stages) circadian preference usually show similar patterns^8,12^, with some evidence of higher percentage of stage 1 sleep in men with a morning, as compared to men with an evening preference^8^. The circadian preferences types also differ from each other in behavioral aspects. As compared to morning preference, a circadian preference towards eveningness has been associated, for instance, with better learning and cognitive abilities^13–15^, but with higher risk for behavioral problems during adolescence^16–19^, poorer mental health and higher prevalence of clinical depression during adulthood^20–22^ and multiple other health risks ^23–29^.

These behavioral aspects related to learning, cognitive function and mental health are also associated with individual variation in sleep EEG characteristics, of which specifically sleep spindles have raised considerably attention lately^30^. Sleep spindles are brief bursts of rhythmic thalamocortical oscillations seen in sleep electroencephalography (EEG) mainly during stage 2 of non-rapid eye movement (NREM) sleep at the sigma frequency range of 10–16 Hz^31,32^. They are a common marker for defining the stage 2 NREM sleep^31^. Spindles can be divided into slow (<13 Hz) and fast (>13 Hz) spindles^33,34^. These two spindle types differ temporally and in topographic distribution, fast spindles occurring earlier and predominating in central brain areas, while slow spindles emerge later and predominate in frontal brain areas^35,36^. Slow and fast spindles have also been suggested to differ etiologically^34^ as well as functionally regarding memory-processes^36–38^. Spindles have been associated with sleep maintenance function by blocking external stimuli processing during sleep^39,40^, general intelligence^41–43^, and they are a key candidate mechanism for memory consolidation during sleep^32,37,44–48^. Changes in spindle characteristics, e.g. decreases in spindle number, density, duration or amplitude, are seen in neurodegenerative and psychiatric diseases^49^, such as social anxiety disorder^50^, Alzheimer’s disease and Mild Cognitive Impairment^42,51^, schizophrenia^52–54^ and autism^55^. Among healthy adults, individual night to night variation in spindle characteristics is low^56–58^, influenced by a high, 96% heritability estimate^59^. There are, however, significant inter-individual, age- and sex-based variation in spindle characteristics^42,57,58,60–66^.

Both individual circadian preference and sleep spindles are considered to be trait-like characteristics of individuals, and both are associated with cognitive function and mental health outcomes. Major interest in spindle research thus far has been in cognitive functioning^67^, and less attention has been paid to the association between spindles and other trait characteristics, such as circadian preference. Yet, as previous studies have shown already significant differences between the circadian types in homeostatic sleep regulation^8–11^, it would be of interest to study whether differences are seen also in spindle characteristics, specifically due to their role in maintaining sleep^32,33^. Regarding previous studies, findings on 24 subjects indicate a higher low (12–14 Hz) sigma power^8^ and a steeper increase of low sigma power during the first three sleep cycles, among those with a morning as compared to those with an evening preference^9^. Sigma power may be considered as an indirect index of sleep spindles, which does not measure directly phasic spindle activity, however^68^. The latter has not yet been assessed and compared between subjects with different circadian preferences. Consequently, this study explores for the first time whether spindle characteristics are related to circadian preference, and whether this relation is moderated by spindle location in frontal and central derivations, frequency (slow and fast spindles), and participant sex, commonly associated with spindles^59,60,62^. Additionally, we explored whether circadian preference is associated with the time distribution of spindle occurrence across the night, and whether any of the significant results regarding circadian preference were moderated by time. We examined these questions among 170 adolescents belonging to a community cohort.

## Results

### Circadian preference, health-related, general sleep and other background characteristics

Of the girls and boys, 14.9% and 14.5% (N = 15, N = 10) had circadian preference towards morningness, 58.4% and 60.9% (N = 59, N = 42) had an intermediate circadian preference and 26.7% and 24.6% (N = 27, N = 17) had circadian preference towards eveningness, respectively. As presented in Table 1, there were no significant differences between the circadian preference groups in sex distribution or in health-related and other background characteristics (P-values > 0.08), or with regard to polysomnography-based sleep stage architecture (P-values > 0.09).

**Table 1:** Descriptive statistics and polysomnography-based sleep characteristics by circadian preference.

	Circadian preference			
	Morning (N = 25)	Intermediate (N = 101)	Evening (N = 44)	P^b^
	Mean ± SD/%	Mean ± SD/%	Mean ± SD/%	
Sex				0.9
*Girls*	60.0	58.4	61.4	
*Boys*	40.0	41.6	38.6	
Age	16.9 ± 0.1	16.9 ± 0.1	16.9 ± 0.1	0.7
Highest education of the parents (SES)				0.08
*Secondary or less*	8.0	13.9	0	
*Vocational*	28.0	18.8	18.2	
*University degree*	64.0	67.3	81.8	
BMI^a^	21.9 ± 2.7	22.1 ± 3.4	22.1 ± 3.5	0.9
PDS	3.4 ± 0.4	3.3 ± 0.4	3.3 ± 0.4	0.6
Sleep duration (hh:mm)^c^	7:33 ± 1:09	7:40 ± 1:11	7:39 ± 1:07	0.9
Wake after sleep onset (WASO)^c^ (hh:mm)	0:31 ± 0:31	0:35 ± 0:26	0:29 ± 0:14	0.5
REM duration (hh:mm)^c^	1:38 ± 0:27	1:37 ± 0:30	1:35 ± 0:30	0.9
REM percentage^c^	20.2 ± 4.7	19.4 ± 5.1	19.3 ± 5.0	0.7
NON-REM duration (hh:mm)^c^	5:54 ± 0:55	6:02 ± 0:42	6:03 ± 0:52	0.7
NON-REM percentage^c^	73.2 ± 6.1	73.3 ± 5.2	74.7 ± 5.1	0.4
Stage 1 duration (hh:mm)^c^	0:52 ± 0:20	0:51 ± 0:23	0:46 ± 0:21	0.2
Stage 1 percentage^c^	11.2 ± 4.1	10.2 ± 4.3	9.4 ± 4.3	0.09
Stage 2 duration (hh:mm)^c^	3:00 ± 0:43	3:11 ± 0:42	3:11 ± 0:40	0.4
Stage 2 percentage^c^	37.2 ± 7.1	38.3 ± 6.3	39.2 ± 6.2	0.4
Stage 3 duration (hh:mm)^c^	2:00 ± 0:34	2:01 ± 0:26	2:06 ± 0:27	0.5
Stage 3 percentage^c^	24.8 ± 6.1	24.8 ± 6.0	26.2 ± 6.2	0.4

^a^BMI refers to Body Mass Index; PDS refers to Pubertal Development Scale. ^b^There were no significant differences in these variables by circadian preference type in oneway-ANOVA or chi square. ^c^All P-values > 0.05 in testing the mean differences between circadian preference groups with one-way ANCOVA, age and sex as covariates.

### General sleep and spindle characteristics by sex

As presented in Table 2, girls had longer duration of total sleep (P = 0.02), REM sleep (P = 0.01) and stage 2 sleep (P = 0.005) as well as stage 2 percentage (P = 0.02) than boys in the independent sample T-test. Regarding spindle characteristics, girls had significantly higher fast spindle amplitude in central derivation (P < 0.0001), fast spindle density in central derivation (P = 0.0007), fast spindle duration in frontal derivation (P = 0.001), and fast spindle intensities in both central (P < 0.0001) and frontal derivations (P = 0.01), but lower slow spindle duration in central derivation (P < 0.003) than boys (Table 2).

**Table 2:** Polysomnography-based sleep and spindle characteristics by sex.

	Girls (N = 101)	Boys (N = 69)	P^a^
	Mean ± SD	Mean ± SD	
Sleep duration (hh:mm)	7:50 ± 1:05	7:24 ± 1:14	0.02
Wake after sleep onset (WASO) (hh:mm)	0:31 ± 0:23	0:35 ± 0:26	0.3
REM duration (hh:mm)	1:42 ± 0:28	1:30 ± 0:30	0.01
REM percentage	20.1 ± 4.7	18.6 ± 5.5	0.06
NON-REM duration (hh:mm)	6:08 ± 0:48	5:54 ± 0:58	0.09
NON-REM percentage	73.5 ± 5.3	73.8 ± 5.3	0.7
Stage 1 duration (hh:mm)	0:48 ± 0:21	0:53 ± 0:22	0.2
Stage 1 percentage	9.6 ± 4.2	10.9 ± 4.3	0.05
Stage 2 duration (hh:mm)	3:18 ± 0:39	2:59 ± 0:45	0.005
Stage 2 percentage	39.3 ± 5.8	37.1 ± 6.6	0.02
Stage 3 duration (hh:mm)	2:02 ± 0:27	2:02 ± 0:28	0.9
Stage 3 percentage	24.6 ± 5.8	25.9 ± 6.2	0.2
Slow spindle amplitude in central	26.1 ± 5.5	25.6 ± 5.2	0.6
Slow spindle amplitude in frontal	24.1 ± 5.6	23.9 ± 4.8	0.8
Fast spindle amplitude in central	23.1 ± 5.7	18.9 ± 4.7	0.000002
Fast spindle amplitude in frontal	15.5 ± 3.9	14.6 ± 3.5	0.1
Slow spindle density in central	0.5 ± 0.2	0.5 ± 0.3	0.4
Slow spindle density in frontal	1.0 ± 0.4	1.0 ± 0.4	0.5
Fast spindle density in central	0.8 ± 0.4	0.6 ± 0.3	0.0007
Fast spindle density in frontal	0.6 ± 0.4	0.5 ± 0.4	0.2
Slow spindle duration in central	1.3 ± 0.1	1.4 ± 0.1	0.003
Slow spindle duration in frontal	1.4 ± 0.08	1.3 ± 0.08	0.6
Fast spindle duration in central	1.4 ± 0.09	1.4 ± 0.1	0.09
Fast spindle duration in frontal	1.4 ± 0.08	1.3 ± 0.09	0.001
Slow spindle intensity in central	34.3 ± 8.1	35.2 ± 8.8	0.5
Slow spindle intensity in frontal	32.6 ± 8.0	32.0 ± 7.0	0.6
Fast spindle intensity in central	32.7 ± 8.6	26.4 ± 7.7	0.000004
Fast spindle intensity in frontal	21.0 ± 5.8	19.0 ± 4.2	0.01

^a^P-values in T-test. SD refers to standard deviation.

### Spindle characteristics by circadian preference

As presented in Table 3, a higher continuous short-MEQ sum score, indicating a preference towards morningness was associated with lower spindle amplitude and intensity (for both P = 0.03) in linear mixed model analyses, adjusted for sex and age. When examined separately as dummy-coded circadian preference categories, the morning preference group differed significantly from the intermediate (for amplitude P = 0.001 and for intensity P = 0.0006) and evening preference group (for both P = 0.01).

**Table 3:** Results from mixed model analysis for spindle characteristics by circadian preference type.

Spindle characteristics	Main effect		Circadian preference and sex interaction	Circadian preference and spindle location interaction	Circadian preference and spindle frequency interaction	Circadian preference and spindle location and frequency interaction
	Estimate (95 % CI)	P	P	P	P	P
**Amplitude (µV)**						
Short MEQ sum	−0.2 (−0.4 ± −0.03)	0.03	0.6	1.8 × 10^−17^	7.0 × 10^−62^	0.2
Morning vs. Intermediate	−3.0 (−4.8 ± −1.2)	0.001	0.3	0.6	0.004	0.03
Morning vs. Evening	−2.6 (−4.7 ± −0.6)	0.01	0.9	0.6	0.08	0.3
Evening vs. Intermediate	−0.4 (−1.8 ± 1.1)	0.6	0.3	0.8	0.3	0.3
**Density (number of spindles per 60 s)**						
Short MEQ sum	0.003 (−0.02 ± 0.03)	0.8	0.5	6.6 × 10^−65^	5.7 × 10^−53^	0.04
Morning vs. Intermediate	0.06 (−0.2 ± 0.3)	0.6	0.8	0.5	0.9	0.04
Morning vs. Evening	0.07 (−0.2 ± 0.3)	0.6	0.5	0.6	0.9	0.1
Evening vs. Intermediate	−0.004 (−0.2 ± 0.2)	0.9	0.2	0.9	0.9	0.8
**Duration (s)**						
Short MEQ sum	−0.0005 (−0.003 ± 0.002)	0.7	0.3	0.00003	0.0005	0.1
Morning vs. Intermediate	−0.03 (−0.05 ± 0.001)	0.06	0.7	0.7	0.1	0.2
Morning vs. Evening	−0.01 (−0.04 ± 0.02)	0.4	0.9	0.6	0.2	0.3
Evening vs. Intermediate	−0.01 (−0.04 ± 0.008)	0.2	0.4	0.8	0.9	0.9
**Intensity (duration (second) multiplied by amplitude, µV)**						
Short MEQ sum	−0.3 (−0.6 ± −0.02)	0.03	0.4	3.8 × 10^−19^	3.8 × 10^−43^	0.2
Morning vs. Intermediate	−4.6 (−7.2 ± −2.0)	0.0006	0.4	0.5	0.006	0.04
Morning vs. Evening	−3.8 (−6.7 ± 0.9)	0.01	0.8	0.4	0.07	0.3
Evening vs. Intermediate	−0.8 (−2.9 ± 1.3)	0.4	0.5	0.7	0.4	0.3

Spindle location (central, frontal) moderated the significant associations between circadian preference and spindle amplitude and intensity when using the continuous MEQ score in two-way interactions in mixed model analyses (P < 0.0001, Table 3). Spindle frequency (slow, fast) moderated the significant associations between circadian preference and spindle amplitude and intensity when using the continuous MEQ score and the dummy-coded circadian preference categories in two-way interactions in mixed model analyses (for the continuous MEQ score P < 0.0001 for both amplitude and intensity and for the morning preference as compared to intermediate preference group P = 0.004 for amplitude and P = 0.006 for intensity, Table 3). The three-way interaction between circadian preference, location and frequency was significant regarding amplitude and intensity for morning as compared to intermediate preference group (for amplitude P = 0.03 and for intensity P = 0.04). There were no significant main effects between circadian preference and spindle density or duration (all P ≥ 0.06).

Table 4 present the post-hoc comparisons in spindle amplitude and intensity between the circadian preference groups using ANCOVA with sex and age as covariates. Circadian preference groups differed significantly regarding the slow spindle amplitude in central (P = 0.0003) and frontal (P = 0.006) derivations. In the pairwise comparisons, the morning preference group had significantly lower slow spindle amplitudes in central and frontal derivations than both intermediate (P < 0.0001 for central and P = 0.001 for frontal) and evening (P = 0.002 for central and P = 0.02 for frontal) preference groups. There were no significant associations between circadian preference groups and fast spindle amplitudes in central or frontal derivations (P = 0.5 and P = 0.2 respectively).

**Table 4:** ANCOVA results for spindle amplitude and intensity by circadian preference type.

Spindle characteristics	Morning	Intermediate	Evening	one-way ANCOVA by circadian preference type	
	Mean (95 % CI)	Mean (95 % CI)	Mean (95 % CI)	F	P
**Amplitude (µV)**					
Slow in central	21.6 (19.3 ± 23.8)	26.8 (25.7 ± 27.8)	26.1 (24.4 ± 27.7)	(2,145) = 8.5	0.0003
Slow in frontal	21.0 (18.9 ± 23.0)	24.7 (23.7 ± 25.7)	24.0 (22.5 ± 25.6)	(2,165) = 5.3	0.006
Fast in central	20.0 (17.7 ± 22.3)	21.5 (20.4 ± 22.5)	21.3 (19.6 ± 23.0)	(2,148) = 0.7	0.5
Fast in frontal	13.9 (12.4 ± 15.3)	15.3 (14.6 ± 16.1)	15.3 (14.2 ± 16.4)	(2,165) = 1.7	0.2
**Intensity (duration (seconds) multiplied by amplitude, µV)**					
Slow in central	27.9 (24.3 ± 31.4)	36.0 (34.3 ± 37.7)	35.1 (32.5 ± 37.6)	(2,145) = 8.5	0.0003
Slow in frontal	27.8 (24.9 ± 30.8)	33.5 (32.1 ± 34.9)	32.1 (29.9 ± 34.3)	(2,165) = 5.9	0.003
Fast in central	28.0 (28.7 ± 31.6)	30.5 (28.7 ± 32.0)	29.8 (27.3 ± 32.4)	(2,148) = 0.7	0.5
Fast in frontal	18.4 (19.5 ± 20.4)	20.6 (19.5 ± 21.6)	20.1 (18.5 ± 21.6)	(2,165) = 1.8	0.2

**Figure 1:**
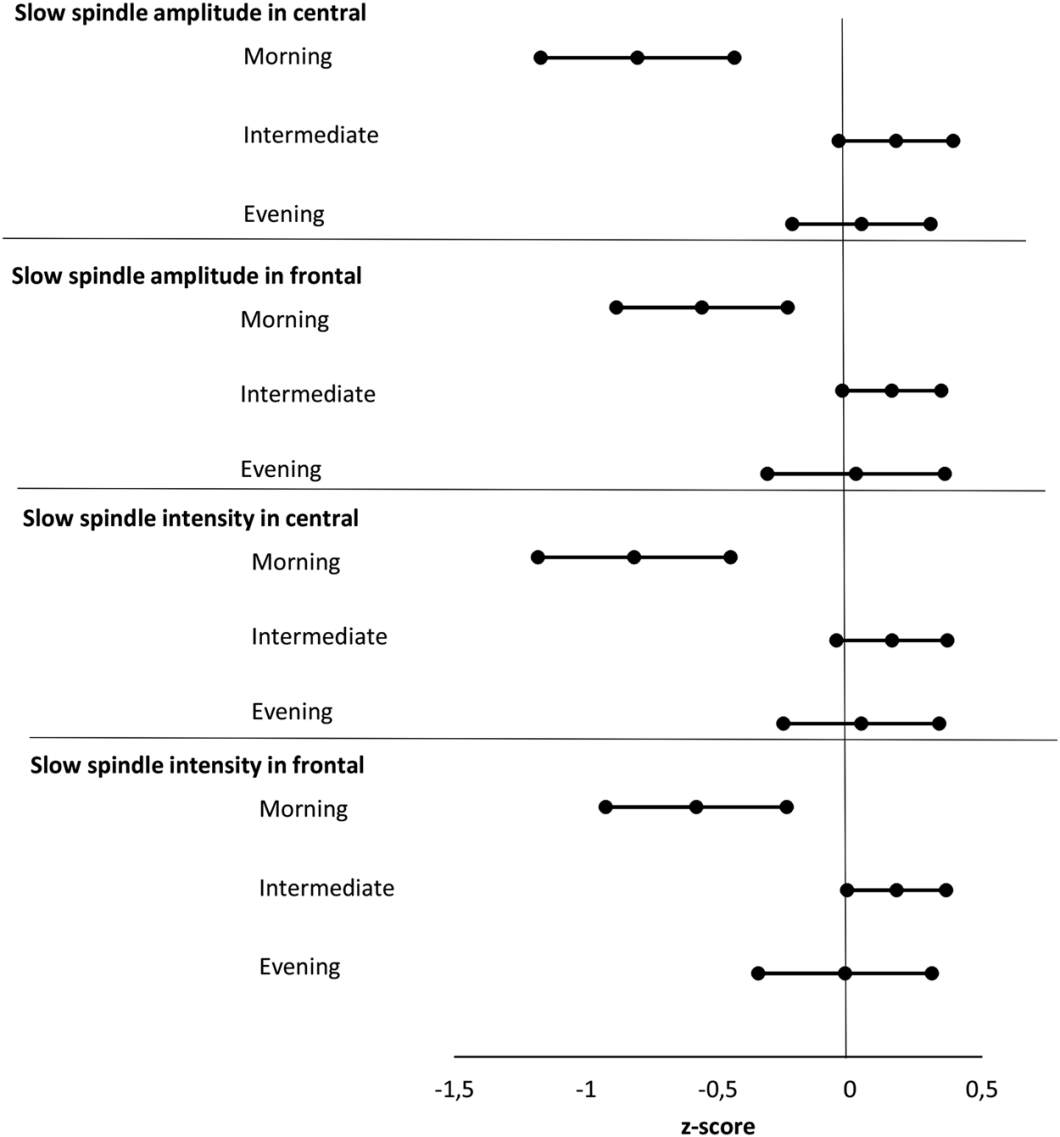
Forest plot for the slow spindle amplitude and intensity in central and frontal derivations by circadian preference group presenting mean and 95 % confidence intervals as z-scores adjusted with sex and age.

The slow spindle intensities differed significantly between circadian preference groups in both central (P = 0.0003) and frontal (P = 0.003) derivations (Table 4). In the pairwise comparisons, the morning preference group had lower slow spindle intensity at both central and frontal derivations as compared to intermediate (P < 0.0001 for central and P = 0.0008 for frontal) or evening (P = 0.002 for central and P = 0.02 for frontal) preference groups. There were no significant associations with circadian preference groups and fast spindle intensities in central or frontal derivations (P = 0.5 and P = 0.2 respectively). We found no significant associations in post-hoc ANCOVAs for spindle duration and density between the circadian preference types (P > 0.08). We illustrate all the significant findings in Fig. 1, showing slow spindle amplitudes and intensities presented as z-scores by circadian preference group.

### Spindle characteristics by circadian preference and sex interaction

As Table 3 shows, sex did not moderate any of the association between short-MEQ sum score or circadian preference groups and spindle characteristics in linear mixed model analysis (all P-values for sex and circadian preference interactions P > 0.2).

### Time distribution of spindles across the night by circadian preference

As an additional post-hoc test for the significant finding between slow spindle amplitude and intensity and circadian preference, we tested whether the time-based distribution of slow spindles across the entire night varied between the circadian preference groups. For these analyses, we chose all the spindles detected in F3, F4, C3 and C4 derivations, and used the time of each spindle occurrence from seconds from the individual sleep onset time as independent variable in unconditional growth models, controlling for age, sex and derivation, and with intercept and time as random-effect variables. We tested the effect of time on slow spindle amplitude and intensity both in linear, and non-linear (quadratic and cubic) mixed models. Linear (estimate = −0.19, Standard Error (SE) = 0.06, P = 0.004) growth models were statistically significant in predicting a decreasing spindle amplitude as a function of (standardized) time parameter. However, higher order non-linear parameters, quadratic (P < 0.0001) and cubic (P = 0.001), resulted in better fit than linear term for the model according to the Akaikes information criterion (AIC). With regard to the spindle intensity, only the cubic time parameter was statistically significant (P = 0.002; P = 0.29 for the linear, and P = 0.70 for the quadratic term). We thus retained the non-linear cubic time parameters in the subsequent models.

Next, we explored whether the circadian preference groups moderated the associations between time, spindle amplitude and intensity by adding interaction terms ‘cubic time parameter* circadian preference group’ to the model. The results showed a significant interaction between cubic time parameter and circadian preference in predicting spindle amplitude, with statistically significant interactions contrasting morning vs. intermediate (P = 0.004) and morning vs. evening (P = 0.004; P = 0.92 for intermediate vs. evening) preference. None of these interactions were further moderated by derivation (P > 0.15).

**Figure 2:**
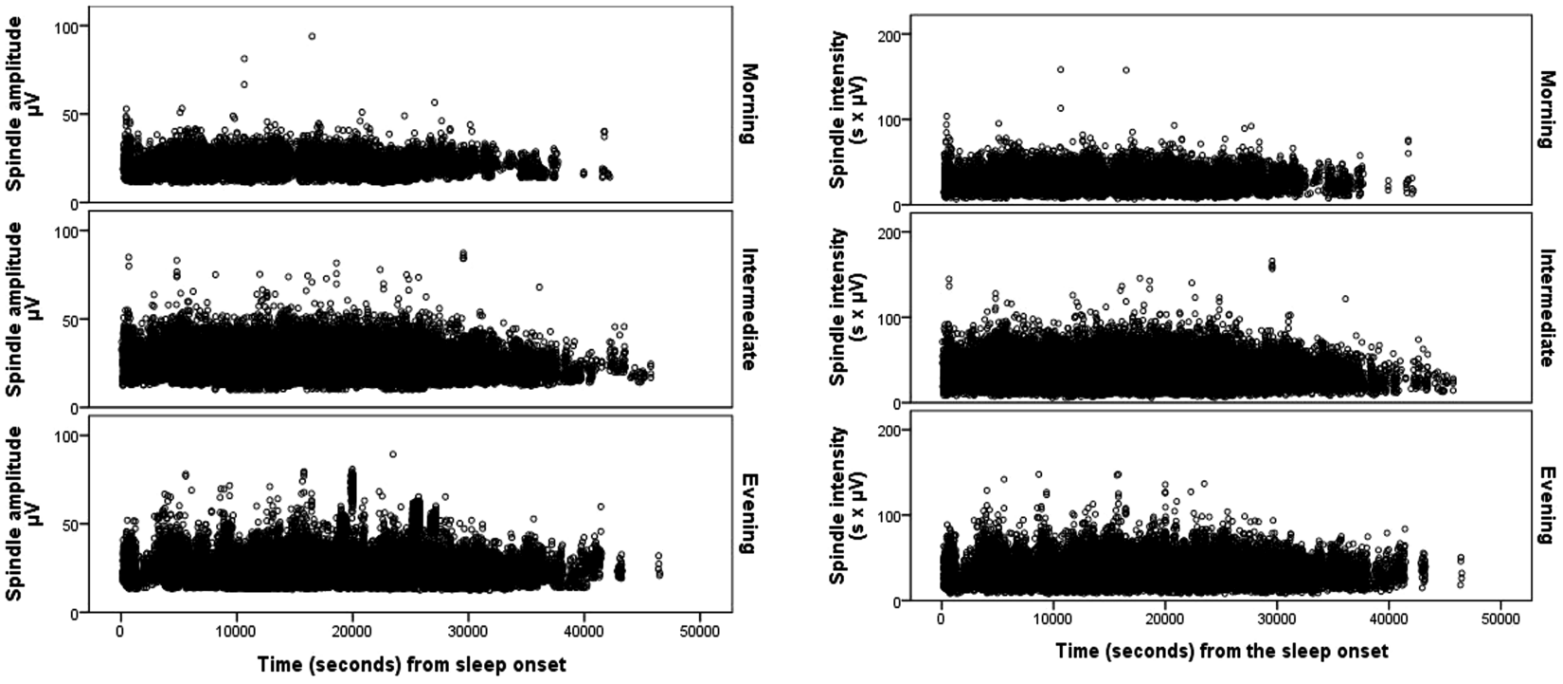
Spindle amplitude (left panel) and intensity (right panel) as a function of time from sleep onset by circadian preference group. Each dot represents a spindle.

In terms of the spindle intensity, significant interactions were found between cubic time parameter and circadian preference (P < 0.0001 for morning vs. intermediate, P = 0.02 morning vs. evening; P = 0.13 intermediate vs. evening preference). Derivation moderated these associations (P < 0.02 in interactions ‘circadian preference*cubic time parameter*derivation’), the contrast between morning vs. intermediate remaining significant in both frontal (P = 0.007) and central (P = 0.03) derivations, and between morning vs. evening only in the central derivation (P = 0.006, P = 0.27 in frontal). The contrast between intermediate and evening became significant in frontal (P = 0.012) but not in central (P = 0.32) derivation. Figure 2 illustrates the spindle amplitude (left panel) and the spindle intensity (right panel) as function of time and circadian preference, such that all derivations are pooled.

## Discussion

Our study is the first to report differences in spindle characteristics and their timing across different circadian preferences. The general pattern of the results indicated that specifically individuals with a circadian preference towards morningness differed from those with a later circadian preference in spindle characteristics. The pattern was particularly evident for slow spindle amplitude and intensity variables both at central and frontal derivations. The contrast was sharpest between morning and intermediate groups, but significant also between morning and evening groups. We also showed, that the distribution of the spindles across the night differed according to the circadian preference. Specifically, morning preference group showed first less increase towards midnight and then earlier decline in spindle amplitude and intensity towards morning as compared to other groups. In terms of spindle density and duration, no significant main effects were observed by circadian preference. Further, even though our results indicated significant sex differences in spindle characteristics, sex did not moderate any of the significant associations.

The current study thus showed that the association between lower slow sleep spindle amplitude and intensity and earlier circadian preference can be detected at the age of 17. As observed in our previous study within the same sample^19^, the circadian preference is reflected in the actual sleep midpoint over eight-day period, which was one hour and 25 minutes earlier in the morning preference group as compared to the evening preference group.

Recent reports provide an increasing insight into the role of the circadian pacemaker in determining certain aspects of sleep EEG oscillations. One important example indicating circadian role in homeostatic sleep regulation is the circadian-dependent slow wave sleep incidence, amplitude, frequency and slope^69^. Another example of this is the effect of circadian clock gene PER3 polymorphism on sleep EEG slow wave activity, where PER3^5/5^ variant found more common among morning preference types is associated more strongly with sleep timing and elevated sleep EEG slow wave activity than PER^4/4^ variant, which is found more common among evening preference types^70^. Our report is a new piece of evidence, suggesting that circadian preference not only provides an optimal framework for sleep timing^71^, but is associated with different sleep spindle phenotypes as well. While we did not find statistically significant differences in the actual sleep duration across the circadian preference groups, previous studies suggest shorter sleep duration among morning-types as compared to other circadian preference types in adulthood^2,25,72^. One could thus hypothesize whether spindle characteristics would explain the shorter sleep duration in morning preference group, as spindles promote sleep by inhibiting thalamic information processing of external sensory stimuli during sleep^39,40^. Intriguingly, our results supported this hypothesis, by showing that especially in the morning preference group, the amplitude and intensity of the sleep spindles showed a flattened increase during the night and earlier declined towards morning faster than in other preference groups. In a developmental perspective, it is intriguing to question whether the old age related decline in the overall spindle activity^67^ actually corresponds with the propensity to shift to a more morning – oriented sleep rhythm along the old age^4^. Previous studies also support this hypothesis of shorter circadian period among morning types as compared to other circadian preference types by reporting faster decay rate of slow-wave activity resulting faster dissipation of sleep pressure among those with morning preference as compared to those with evening preference^8–10^.

Morning preference types have also been shown to have higher slow sigma activity than evening types in NREM sleep in the study of Mongrain *et al*.^8^. This finding might be partially contradicting our finding on lower slow spindle amplitudes and intensities among morning vs. other circadian preference groups. However, in another study of theirs, it is shown that morning types have a steeper increase in low sigma power during the first three sleep cycles, but then, a significant decrease during the last cycle at parietal derivation as compared to evening types^9^. This finding regarding the steeper decrease during the last cycle shows a similar trend than our results from the time distribution analyses during the night. Although, it has to be acknowledged that the sigma power is only indirect measure of sleep spindles^68^.

Previous studies have associated both lower spindle activity and earlier circadian preference with lower scores in cognitive ability tests^13–15,73^. Our observation now connects for the first time circadian preference directly with spindle characteristics in a way that is coherent with these earlier observations. However, in contrast to our findings here, lower spindle amplitude has been associated with increased risk for psychiatric diseases^52,53^, which on the other hand, have usually been associated with circadian preference towards eveningness^20–22,73^. Yet, no distinction between slow and fast spindles were made in these studies^52,53^, and in that sense they are not comparable to our findings. The difference in slow and fast spindle associations with disorders are seen for instance regarding rapid eye movement sleep behavior disorder, where slow spindle densities are found to be increased, while fast spindles are decreased^74^.

In accordance with previous studies^60,61,63^, we found that sex was associated with spindle characteristics, such that girls had in central derivation higher fast spindle amplitude in central derivation and fast spindle density, fast spindle duration in frontal derivation and fast spindle intensities in both central and frontal derivations than boys. Previous studies^63,75^ have also reported higher spindle densities among females as compared to males. However, in contrast to these other studies, we also found higher intensities regarding fast spindles among girls as compared to boys.

The strengths of our study include the relatively large sample size, with a homogeneous age distribution. This increases the reliability of our analyses, as spindles are rather dependent on age^67^, presenting usually a major confounding factor. While the observed spindle densities were somewhat low for some derivations^30,35^, which might be due to the fact that we had a proper control of derivation impedance both at the target and reference electrodes. We also limited the duration of the spindle to 250 ms on both directions from the peak maximum to avoid artefacts. Finally, the participants slept according to their own schedule, without any forced sleep times.

As limitations, we used here for the assessment of circadian preference the shortened 6-item version of the original 19-item MEQ^3^. However, the shortened version has shown good reliability in the assessment of circadian preference, explaining 83% of the variance in entire scale^76^. It is to be noted however, that the use of 19-item MEQ would give even stronger reliability in assessment of circadian preference and could possibly further strengthen the results we report here. Second, the strict age distribution limited to adolescents diminish external validity to other age groups. Third, there exists several automated spindle detection algorithms. While the Ferrarelli method^52,77^ used in this study is widely applied, there is still lack of cross-validation studies across different methods^34^.

In conclusion, our study is the first to report differences between circadian preference types in the overnight spindle characteristics. Our results indicated lower slow spindle amplitude and intensity among morning preference group as compared to other circadian preference groups. Additionally, the morning preference group showed faster decline in spindle amplitude and intensity towards morning as compared to other groups. It is possible that differences in spindle characteristics are associated with circadian period differences between circadian preference types or, as a new intriguing hypothesis, underlie the previously reported behavioral and cognitive differences between the circadian preference types. However, our study is aimed to be an explorative opening for these questions. It has to be acknowledged, that more studies are needed to confirm the results and to bring more insight into the mediating mechanisms.

## Methods

### Participants

The data of this study are based on an urban community-based cohort comprising initially 1049 healthy singletons born between March and November 1998 in Helsinki, Finland. Two first sleep follow-ups were conducted during years 2006 and 2009–2011, at 8 and 12 years of age on average. The details of the cohort are described in more detail in previous reports^78–80^. In years 2014–15, all the cohort members who participated in the previous follow-up at age 12, and who lived within the 30 km radius from Helsinki (N = 279, 77.1% of the participants of the previous follow-up) were invited to a follow-up. Of them, 197 (70.6%) participated at the age of 17. The analytic sample comprised 170 (101 females and 69 males, mean age = 16.9, SD = 0.1 years, range from 16.6 to 17.2 years) adolescents who had complete records of circadian preference and an overnight sleep EEG measurement.

The analytic sample of 170 participants in this study did not differ significantly from the rest of the participants in the initial cohort (N = 879) or from the rest who were invited to the follow-up but did not participate (N  = 109) regarding mother’s age or Body Mass Index (BMI) at birth, gestational age, birthweight, length at birth or maternal alcohol or licorice consumption during pregnancy (all P > 0.05) in t-tests. The Ethics Committee for Children and Adolescents’ Diseases and Psychiatry at the Helsinki University Central Hospital approved the study protocol. All methods were performed in accordance with the relevant guidelines and regulations. All participants and their parents gave their written informed consent.

### Assessment of the circadian preference

Participants filled the Horne-Östberg Morningness-Eveningness Questionnaire (MEQ) that measures the personal timing preference of the intrinsic circadian period^3^. We used a shortened 6-item version of the scale to assess the individual circadian preference (consisting the items 4, 7, 9, 15, 17 and 19 from the original MEQ), as it is reported to explain 83% of the variance in the sum of the entire 19-item scale^76^. The sum score ranged from 5 (Evening preference group) to 27 (morning preference group). The scale yields three circadian preference types: the definite or moderate morning preference (19 to 27 points), the intermediate preference (13 to 18 points), and the definite or moderate evening preference (5 to 12 points), reflecting the original MEQ sum score scaling^3,76^. The test-retest reliability of MEQ with 2 month interval is reported to be around 0.89^81^. We have previously shown in this same study sample that Evening preference group had statistically significant later actigraphy-based midpoint of sleep as compared to the other circadian preference groups^19^.

### Sleep EEG recording

Overnight polysomnographic sleep recordings (PSG) were conducted in the homes of the participants with SOMNOscreen plus (SOMNOmedics GmbH, Germany). Electroencephalography (EEG) measurements were recorded with gold cup electrodes at 6 EEG locations (F3, F4, C3, C4, O1 and O2) and two derivations for the mastoids (A1, A2) according to the standardized 10/20 system. The electro-oculogram (EOG) and the electromyogram (EMG) were measured by using disposable adhesive electrodes (Ambu Neuroline 715, Ambu A/S, Denmark), two locations for EOG and three locations for EMG. In addition, an online reference Cz and a ground electrode in the middle of forehead were used. The sampling rate was 256 Hz (the hardware filters for SOMNOscreen plus are 0.2–35 Hz).

All signals were digitally offline filtered with pass band of 0.5–40 Hz (Hamming windowed sinc zero-phase FIR filter, cut-off (−6 dB) 0.25 Hz and 44.3 Hz respectively) and re-referenced to the average signal of A1 and A2 electrodes. Sleep stages from PSG data were scored manually with the DOMINO program (v2.7; SOMNOmedics GmbH, Germany) by three experienced researchers in 30-second epochs.

### Spindle Analysis

**Figure 3:**
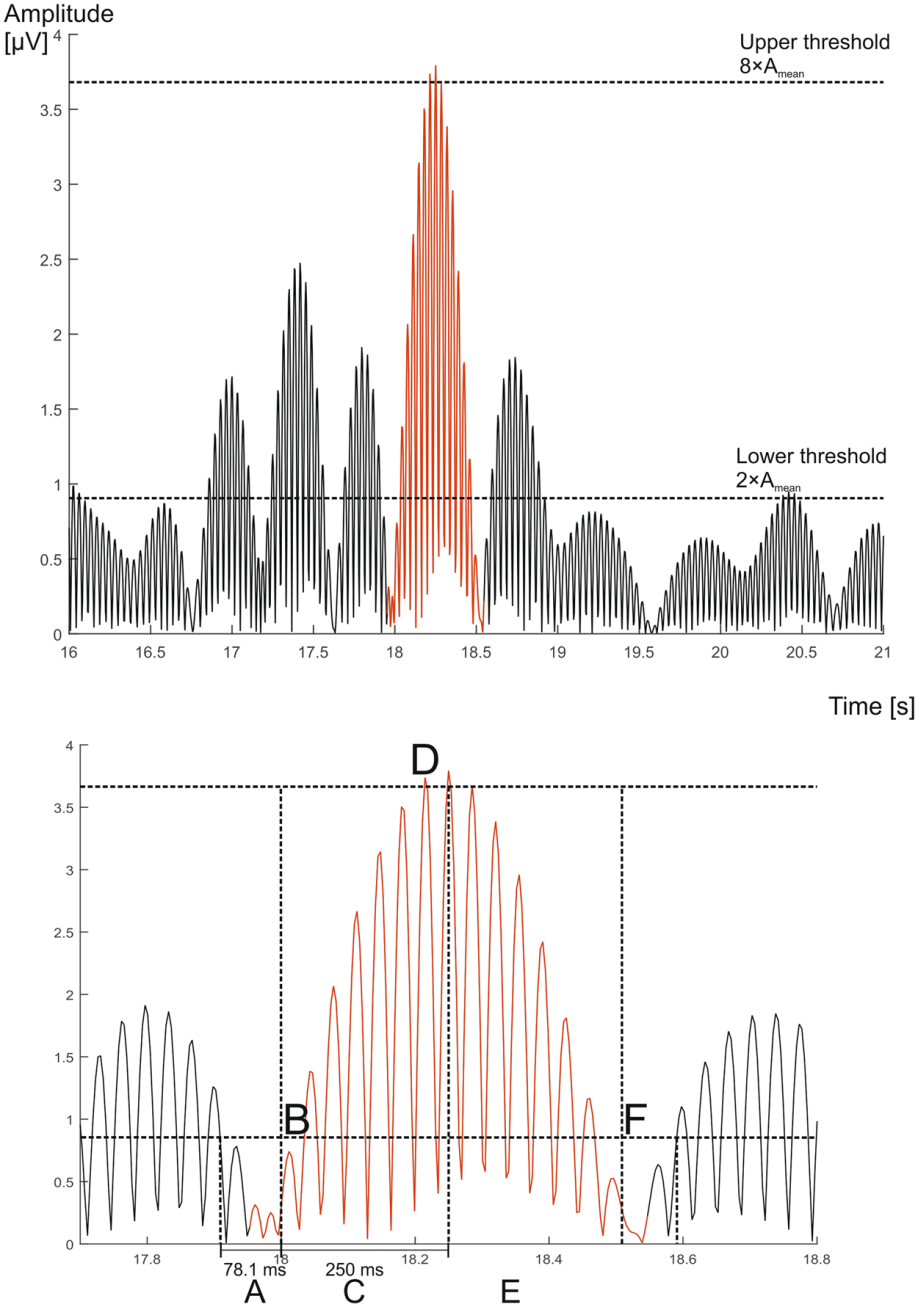
Spindle detection criteria. The rectified EEG signal (**A**) stays below the lower threshold for at least 78.1 ms (20 samples at a sampling rate of 256 Hz), (**B**) crosses the lower threshold (2*mean of channel amplitude). (**C**) Stays above the lower threshold for at least 250 ms without meeting the criterion (**A**,**D**) crosses the upper threshold (8*mean of channel amplitude) at least once and for the duration of at least one sample. The highest amplitude above the threshold is registered as the peak amplitude, where (**E**) after the peak, stays above the lower threshold for at least 250 ms without meeting the criterion (**A**,**F**) recovers back to a level below the lower threshold for 78.1 ms within 2 seconds from the onset (thus duration of a spindle can be 500–2000 ms).

Spindles were computationally extracted with the method described by Ferrarelli^52,77^. The manually scored PSG signals were converted to EDF format in DOMINO software and then further analyzed for spindle detection by using functions of EEGlab 13.5.4b^82^ running on Matlab R2015a (The Mathworks Inc., USA). We extracted spindles from N2 sleep, with a scalp-electrode contact impedance value equal or lower than 10 kΩ during the corresponding 30-second epoch. The spindle analysis was conducted in different frequency bands (10–13 Hz, and 13–16 Hz) in order to differentiate between the slow and fast spindles^34^. The threshold values for finding spindle peak amplitude in each derivation were defined by the mean of the derivation amplitude (µV) multiplied with 2 (lower) and 8 (higher) including all valid epochs (sleep stage N2 and impedance≤10 kΩ). These threshold values have been described by Ferrarelli to be best matching with visual and automatic spindle detection^52^. Thus, we used derivation -wise threshold definitions, taking into account that signals may vary across the derivations. Furthermore, restriction for the spindle duration was set to 250ms on both directions from the peak maximum. Signal amplitude was required to stay under the lower threshold for 78.1 ms which is approximately the duration of one sine period at 13 Hz. This was done in order to prevent false alarms in spindle detection (Fig. 3 illustrates spindle detection criteria). The spindle amplitude, duration, intensity (spindle duration multiplied by spindle amplitude) and density (number of spindles per 60 second epoch) were measured for both at central and frontal derivations, using the averages between the left and right hemisphere derivations respectively. We tested the skewness of our spindle variables and they were for the amplitude, intensity and duration approximately symmetric (between −0.5 and 0.5, for amplitude = 0.4, for intensity = 0.4, for duration = −0.2), but for density highly skewed (skewness 2.8). We thus did a logarithmic transformation for the density variable.

### Sample descriptive variables

Health-related and other background characteristics include body mass index (BMI, kg/m^2^), level of pubertal maturation, socioeconomic status (SES) and actigraph-based sleep duration and wake-after-sleep onset (WASO). BMI was based on the height and weight measurements by a research nurse. Pubertal maturation was self-assessed with the Pubertal Developmental Scale (PDS)^83^. The PDS scale consists of five self-reported items concerning body hair (scored from 1 = no changes yet to 4 = seems fully developed), growth spurts (scored from 1 = no changes yet to 4 = development seems complete), skin changes (scored from 1 = no changes yet to 3 = development seems complete), and menarche (scored 1 = no or 4 = yes) and breast development (scored from 1 = no changes yet to 4 = development seems complete) for girls, and facial hair (scored from 1 = no changes yet to 4 = seems fully developed) and voice change (scored from 1 = no changes yet to 4 = seems completed) for boys. SES was defined as the highest self-reported education level of either parent (classified as (1) secondary or lower, (2) vocational degree, or (3) university degree.

### Statistical analyses

We first analyzed the mean differences between sexes in spindle and general sleep characteristics with independent sample T-test. The distribution of circadian preference by health-related and other background characteristics was tested with two-sided chi-square and one-way ANOVA.

Secondly, we used linear mixed model analysis with a random intercept for studying amplitude, duration, density and intensity of spindles in general in association with short MEQ sum, and dummy-coded morning versus intermediate, morning versus evening and evening versus intermediate -groups, adjusted with age and sex. Linear mixed model analysis for also used to study the moderating effects of sex, spindle location (central, frontal) and frequency (fast, slow) by using two-way and three-way interaction terms. For any significant interactions, we present post-hoc ANCOVAs for the pairwise comparisons between the circadian preference groups. To illustrate the significant findings, we present them in forest plots, with standardized spindle characteristics to facilitate interpretation of the results.

Third, if the linear mixed models indicated a significant association between circadian preference and spindle characteristics, we performed unconditional linear growth curve analyses to study whether the spindle characteristic was associated with time distribution of spindles during the night. The time was calculated as spindle occurrence time as seconds from the individual sleep onset time, thus taking into account individual circadian timing. We modeled the time both as linear, and as in higher-order quadratic (time*time) and cubic (time*time*time) terms. If the time effect was significant in predicting spindle characteristics, we analyzed interaction between circadian preference and time, and between circadian preference, time and derivation in predicting spindle characteristics. We used intercept and time as random-effect variables in these analyses. Estimation method was maximum likelihood and denominator degrees of freedom was corrected using Satterthwaite approximation when calculating the p-values. All analyses were performed with IBM SPSS Statistics 23.0 software.

### Data Availability

The datasets analyzed during the current study are not publicly available due to protecting participant confidentiality but are available from the corresponding author on reasonable request, and with the permission of both the Study Board and the Ethics Committee for Children and Adolescents’ Diseases and Psychiatry at the Helsinki University Central Hospital.
