# Publisher Correction: Circadian preference towards morningness is associated with lower slow sleep spindle amplitude and intensity in adolescents

**DOI:** 10.1038/s41598-018-20335-y

**Published:** 2018-01-23

**Authors:** Ilona Merikanto, Liisa Kuula, Tommi Makkonen, Róbert Bódizs, Risto Halonen, Kati Heinonen, Jari Lahti, Katri Räikkönen, Anu-Katriina Pesonen

**Affiliations:** 1 Department of Psychology and Logopedics, Faculty of Medicine, University of Helsinki, Helsinki, Finland; 2 National Institute for Health and Welfare, Helsinki, Finland; 3 Institute of Behavioural Sciences, Semmelweis University, Budapest, Hungary; 4 Epilepsy Centre, National Institute of Clinical Neurosciences, Budapest, Hungary; 5 Helsinki Collegium for Advanced Studies, Helsinki, Finland

Correction to: *Scientific Reports* 10.1038/s41598-017-13846-7, published online 03 November 2017

The original HTML version of this Article contained a typographical error in the publication date ‘03 November 2017’ which was incorrectly given as ‘06 November 2017’. This has now been corrected in the HTML version of the Article.

